# Accessibility to Parks and Trails and Physical Health Measures in CATSLife: Evaluating Selection

**DOI:** 10.1093/geroni/igaa057.1642

**Published:** 2020-12-16

**Authors:** Chandra Reynolds, Elijah Knaap, Robin Corley, Kyle Gebelin, Elizabeth Munoz, Soo Rhee, Sally Wadsworth

**Affiliations:** 1 University of California, Riverside, Riverside, California, United States; 2 University of California-Riverside, Riverside, California, United States; 3 University of Colorado Boulder, Boulder, Colorado, United States; 4 University of California - Riverside, Riverside, California, United States; 5 The University of Texas at Austin, Austin, United States

## Abstract

Associations of obesity and cardiovascular health with neighborhood walkability have been observed in the literature; however, limited research has evaluated self-selection that may underlie associations. We measured accessibility to parks and trails using geospatial measurements as well as self-reported activity-friendliness of neighborhoods (ease of walking, biking, and recreating) in 1140 participants (ages 28-49 years) in the ongoing Colorado Adoption/Twin Study of Lifespan behavioral development and cognitive aging (CATSLife). Physical health indicators included BMI, resting heart rate, and mean arterial blood pressure. The relative similarity of siblings’ accessibility was evaluated to consider self-selection; all models were adjusted for sociodemographics including education. BMI was associated with accessibility to parks, with each increasing log(mile) distance associated with 1.2 BMI unit increase (se=.49, p<0.02). Self-reported neighborhood activity-friendliness was comparable in prediction of BMI (p<0.01). Greater trail accessibility was associated with lower resting heart rate (b=-.30, se=.14, p<0.04) and mean arterial pressure (b=-0.33, se=0.14, p<0.03), whereas self-reported neighborhood activity-friendliness was not associated (p>0.40). Measures of park accessibility tended to be more similar among identical twins (median ICC = 0.30) than fraternal twins or siblings (median ICC = 0.15) or siblings in adoptive families (median ICC = 0.12), excluding siblings who live together. Measures of trail accessibility were consistent across sibling types (median ICCs = 0.25-0.27). Sibling similarity for park accessibility modestly increased with genetic relatedness suggesting potential heritable contributions, whereas comparable similarity was apparent for trail accessibility. Altogether, small associations were observed for park and trail access with physical health, with indications of environmental selection.

